# Outcomes and prognostic factors for aggressive posterior retinopathy of prematurity following initial treatment with intravitreal ranibizumab

**DOI:** 10.1186/s12886-018-0815-1

**Published:** 2018-06-26

**Authors:** Qizhe Tong, Hong Yin, Mingwei Zhao, Xiaoxin Li, Wenzhen Yu

**Affiliations:** 1 Department of Ophthalmology, Ophthalmology & Optometry Center, Peking University People’s Hospital, Beijing, China; 2 Key Laboratory of Vision Loss and Restoration, Ministry of Education, Beijing, China; 3 Beijing Key Laboratory of Diagnosis and Therapy of Retinal and Choroid Diseases, Beijing, China

**Keywords:** Aggressive posterior retinopathy of prematurity, Retinal detachment, Recurrence, Ranibizumab, Intravitreal injection, Neutrophil count, Postmenstrual age, Birthweight, Retinal hemorrhage

## Abstract

**Background:**

This study sought to identify factors associated with retinal detachment and retreatment of aggressive posterior retinopathy of prematurity (APROP) initially treated with intravitreal ranibizumab (IVR) injection as well as the efficacy of IVR treatment.

**Methods:**

This was a retrospective study. A total of 83 preterm infants (160 eyes) diagnosed with APROP who were primarily treated with IVR were included. The 160 eyes were divided into two groups based on the anatomic outcomes. Group A included 35 eyes that developed retinal detachment, and Group B included 125 eyes without retinal detachment. The following patient factors were retrospectively reviewed: gender, gestational age (GA), birth weight (BW), postmenstrual age (PMA) at first treatment, iris neovascularizations, retinal hemorrhage, neutrophil and lymphocyte counts before the first intravitreal injection, neutrophil-to-lymphocyte ratio (NLR), anatomical outcomes, additional treatment and follow-up time. Three dummy variables were created as dependent variables based on the methods of retreatment. The possible risk factors for APROP were evaluated, and statistical analyses included univariate and multivariate logistic regression.

**Results:**

A total of 160 eyes from 83 preterm infants (56 males and 27 females) underwent initial IVR treatment with a follow-up time of 17.17 ± 10.54 months. Thirty-five of the 160 (21.9%) eyes progressed to retinal detachment, and 82 of the 125 (65.6%) non-retinal detachment eyes needed retreatment, with favorable anatomical outcomes. The disease improved approximately 1.5 ± 1.2 weeks after the first IVR treatment. The mean recurrence period of APROP was approximately 7.5 ± 6.9 weeks after the first IVR treatment. Multiple logistic regression analysis revealed postmenstrual age (*P* < 0.001) and neutrophil count (*P* = 0.009) as the most significant factors for retinal detachment in APROP. Retinal hemorrhage (*P* = 0.007) and BW (*P* = 0.04) were most significantly associated with APROP recurrence and retreatment.

**Conclusions:**

IVR injection is an effective treatment for APROP. In this study, older postmenstrual age and low neutrophil count were identified as risk factors for retinal detachment in APROP. In addition, retinal hemorrhage and low BW were significantly associated with recurrence and retreatment in non-retinal detachment APROP. Thus, patients with a lower BW, older postmenstrual age, low neutrophil count and retinal hemorrhage should be reexamined in a timely and more frequent manner.

## Background

Retinopathy of prematurity (ROP) is a retinal vascular disorder of preterm infants that is a leading cause of childhood blindness [1]. Aggressive posterior retinopathy of prematurity (APROP) is a more virulent form of ROP that is observed in more immature babies with extremely low birth weight (BW); this aggressive form affects zone I or posterior zone II. APROP is characterized by a flat neovascular network at the simple junction between vascularized and nonvascularized retina [2]. Increased dilation and tortuosity of retinal vessels in all four quadrants and intraretinal shunting can be observed in the fundus of APROP patients. APROP does not generally progress through the classic stages 1 to 3 and can quickly lead to total retinal detachment, which causes severe visual impairment or blindness without timely treatment. Late retinal detachment is also a leading cause of blindness in patients with regressed ROP during childhood [3]. Preterm newborns with APROP require effective treatment to avoid retinal detachment, which leads to unfavorable anatomical outcomes and poor sight.

The pathological process of ROP includes two postnatal phases. Phase 1 involves delayed physiological retinal vascular development, and phase 2 involves vasoproliferation [4]. There is also a prephase of antenatal sensitization due to inflammation [5] as inflammation and the innate immune system are related to ROP progression. Neutrophil and lymphocyte counts are relatively easy measures that can be used to assess the inflammatory process, and previous articles have reported that hematological parameters may include unsuspected prognostic information associated with serum components. As such, the role of the neutrophil-to-lymphocyte ratio (NLR) in tumors, diabetes mellitus, heart disease and other diseases has been reported previously, while the effect of neutrophils, lymphocytes and the NLR on ROP has been poorly characterized.

CRYO-ROP (cryotherapy for ROP) and ETROP (early treatment of ROP) studies have demonstrated various satisfactory outcomes after peripheral thermoablation (cryo/laser) of nonvascularized retinas. Cryotherapy was first used clinically to treat acute ROP in the 1980s [6]. The results of the Multicenter Trial of Cryotherapy for Retinopathy of Prematurity support the long-term efficacy and safety of cryotherapy for the treatment of threshold ROP [7]. ROP treatment subsequently shifted slowly from cryotherapy to laser therapy in the 1990s. Laser photoablation of the peripheral retina is an effective treatment for APROP located in zone 1 [8]. A meta-analysis showed that laser therapy for type 1 and threshold retinopathy of prematurity (ROP) may cause more eye complications and higher myopia than anti-VEGF therapy [9]. The role of vascular endothelial growth factor (VEGF) in ROP pathogenesis has been demonstrated previously, and anti-VEGF treatments represent another therapeutic option. Dorta et al. [10] reported the successful treatment of type 1 ROP in a study of twelve consecutive eyes from 7 premature infants using an intravitreal injection of bevacizumab (Avastin). Menke et al. [11] reported that intravitreal ranibizumab monotherapy was an effective treatment for retinopathy of prematurity zone II stage 3 with plus disease. However, serum VEGF was suppressed more in patients with type 1 ROP who received IVB treatment than in those who received IVR treatment [12].

The recognition of risk factors for APROP is useful for the prediction of disease development and timely intervention. The gestational age (GA), BW, hyperoxia and systemic inflammation are important factors that may increase the risk of APROP. The mean GA and BW of infants with APROP were significantly lower than those of infants with non-APROP in a previous study [13]. Moreover, compared with infants with non-APROP, infants with APROP required heavier laser treatment and exhibited a higher retreatment rate.

The present study investigated the efficacy of intravitreal injection of ranibizumab (IVR) for the treatment of APROP and analyzed suspected risk factors associated with tractional retinal detachment and retreatment to identify independent risk factors for the prognosis of APROP.

## Methods

### Study design

In this retrospective study, we reviewed the medical records of preterm infants diagnosed with APROP who received IVR as a first treatment at Peking University Peoples’ Hospital between July 20, 2012, and August 26, 2016. The study was performed in accordance with the principles of the Declaration of Helsinki, 1995 (revised in Edinburgh in 2000), and the Institutional Review Board of our hospital approved the study. The parents of the patients signed a consent form prior to treatment.

ROP was classified according to the International Classification of ROP (2005 revised) [2]. APROP is defined as a flat neovascular network at the simple junction between the posterior vascularized and nonvascularized retina, with increased dilation and tortuosity of retinal vessels in all four quadrants and intraretinal shunting in some cases. Notably, some severe types of ROP, such as zone I any stage ROP with plus disease and zone II stage 2 or 3 ROP with plus disease (termed type 1 ROP, which resembles APROP) need to be differentiated. The following inclusion criteria were used: 1) patients diagnosed with APROP and initially treated with IVR; and 2) follow-up of at least 6 months without retinal detachment or development of tractional retinal detachment within 6 months. The following exclusion criteria were used: 1) follow-up less than 6 months without retinal detachment; 2) history of previous anti-VEGF treatment in another institute; 3) first treatment was not IVR; and 4) patients with fever, infectious disease or leukemia when examined by our ophthalmologists.

The following data were recorded: gender, GA, BW, postmenstrual age (PMA) at first treatment, iris neovascularizations, retinal hemorrhage, neutrophil and lymphocyte counts before the first intravitreal injection, neutrophil-to-lymphocyte ratio (NLR), anatomical outcome, additional treatment and follow-up time.

### Intervention

An ophthalmologist examined all patients preoperatively using binocular indirect ophthalmoscopy. Routine blood examinations were performed for each infant, and anesthesiologists performed preoperative assessments of every patient within 24 h before the intravitreal injection. The infants received tobramycin eye drops in the affected eye four times on the day before surgery and six times on the day of surgery. All infants received IVR in the operating room under topical anesthesia with oxybuprocaine hydrochloride eye drops and general anesthesia with sevoflurane. Povidone iodine was applied using sterile cotton gauze to sterilize the cornea, conjunctival sac, eyelids, and periorbital skin in all patients prior to intravitreal injection. Povidone iodine was flushed onto the ocular surface. A speculum for infants was placed between the lids to prevent contact of the eyelashes and eyelid margins with the injection needle. Sterile gloves and forceps were used during all injections. Ranibizumab (0.3 mg in 0.03 mL) was injected into the vitreous cavity 1.0 mm posterior to the corneal limbus using a disposable 1-mL syringe with a 30 G needle. The speculum was removed after IVR completion, and tactile pressure was determined with the lids closed. Antibiotic eye drops were used four times daily for 1 week after bilateral IVR injections.

The patients were reexamined one day after IVR and weekly or biweekly to monitor disease progression. Our ophthalmologists carefully monitored the patients and evaluated disease regression or recurrence, peripheral vascularization and recurrence of retinal detachment at each follow-up visit. The reappearance of plus disease or the appearance of a ridge was considered recurrence. Regression was defined as plus disease disappearance or continued retinal vessel growth into the previous avascular peripheral area. A second IVR treatment was administered to infants with recurrence of ROP in zone 1 or posterior zone 2. Laser treatment using an 810-nm diode laser was also used in infants with recurrence in the peripheral retina.

All eyes were divided into two groups according to anatomical outcomes. Group A included 35 eyes that developed retinal detachment, and Group B included 125 eyes without retinal detachment. Group B was further classified into 2 subgroups: no recurrence after one IVR treatment and recurrence that required retreatment.

### Statistical analysis

Statistical analyses were performed using the statistical software SPSS version 20.0 (IBM, New York, United States). The Kolmogorov-Smirnov test was used to test the normality of continues variables. Continuous variables subject with normal distribution, such as GA, BW, PMA, and neutrophil and lymphocyte counts before the first intravitreal injection, are expressed as the means ± standard deviation. Medians and interquartile range were calculated for non-normally distributed continuous variables. Three dummy variables were created as dependent variables based on the method of retreatment. First, univariate logistic regression analysis was performed to identify suspected risk factors. Odds ratios (ORs) and 95% confidence intervals (CIs) were calculated for each risk factor. Second, variables exhibiting a significant correlation or a tendency toward an association with retinal detachment or retreatment (*P* < 0.2) in the univariate logistic regression analysis were included in the stepwise logistic regression model. Third, variables included in the multiple logistic regression model were removed if they did not maintain significance at *P* < 0.05.

The predicted probability for retinal detachment and retreatment were computed using multivariate logistic models. Receiver operating characteristic (ROC) curve and area under the curve (AUC) analyses were performed to estimate the predictive capacity of the models. A *P* value less than 0.05 was considered significant, and all *P* values were two-sided throughout the study.

## Results

### Patient demographic data

A total of 160 eyes of 83 patients (56 males and 27 females) were included in the study. Table 1 shows the demographic and clinical characteristics of the infants in the study. Thirteen of the 83 infants were the product of twin pregnancies. The following characteristics were noted: mean GA 30.0 ± 2.0 weeks (range: 26 to 35 weeks), mean BW 1340 ± 334 g (range: 800 to 2300 g), mean PMA 36.6 ± 2.0 weeks (range: 32.29 to 47 weeks), mean follow-up time 17.17 ± 10.54 months (range: 6.00 to 51.00 months), neutrophil count 2.50 ± 1.53*10^9^/L (range: 0.42 to 7.41*10^9^/L), lymphocyte count 4.85 ± 1.50 *10^9^/L (range: 2.18 to 9.01*10^9^/L), and median NLR 0.471 (QAR, 0.307–0.694).

**Table 1:** Patient characteristics

	Total	Retinal detachment	Non-retinal detachment	One injection subgroup	Retreatment subgroup
Eye	160	35	125	43	82
GA (weeks)	30.0 ± 2.0	30.7 ± 2.0	29.8 ± 2.0	30.6 ± 2.3	29.53 ± 2.09
BW (g)	1340 ± 334	1376 ± 374	1330 ± 323	1457 ± 418	1289 ± 296
PMA (weeks)	36.6 ± 2.0	38.3 ± 2.0	36.2 ± 1.8	36.6 ± 2.1	36.09 ± 1.78
Gender (male/female)	56/27	18/9	50/21	20/8	36/15
neutrophil count (*10^9^/L)	2.50 ± 1.53	1.95 ± 1.08	2.66 ± 1.60	2.77 ± 1.56	2.56 ± 1.63
lymphocyte count (*10^9^/L)	4.85 ± 1.50	4.91 ± 1.39	4.84 ± 1.53	4.59 ± 1.52	4.94 ± 1.53
NLR	median 0.47 (QAR 0.31–0.69)	0.37 (0.19,0.60)	0.49 (0.33,0.75)	0.51 (0.36,0.80)	0.480 (0.272,0.698)

### Progress and anatomic outcome

The median period of reexamination after the first injection was 7 days (QAR, 5–10 days). A total of 61.6% of all treated eyes were relieved, while 13.2% of the eyes developed retinal detachment at the first reexamination.

Thirty-five of the 160 eyes developed an unfavorable anatomic outcome (tractional retinal detachment) and required surgery. Forty-three eyes in the non-retinal detachment group regressed after one injection, and 82 of the 125 eyes without retinal detachment regressed after retreatment.

### Subgroup analysis: retinal detachment and no retinal detachment subgroups

**Fig. 1:**
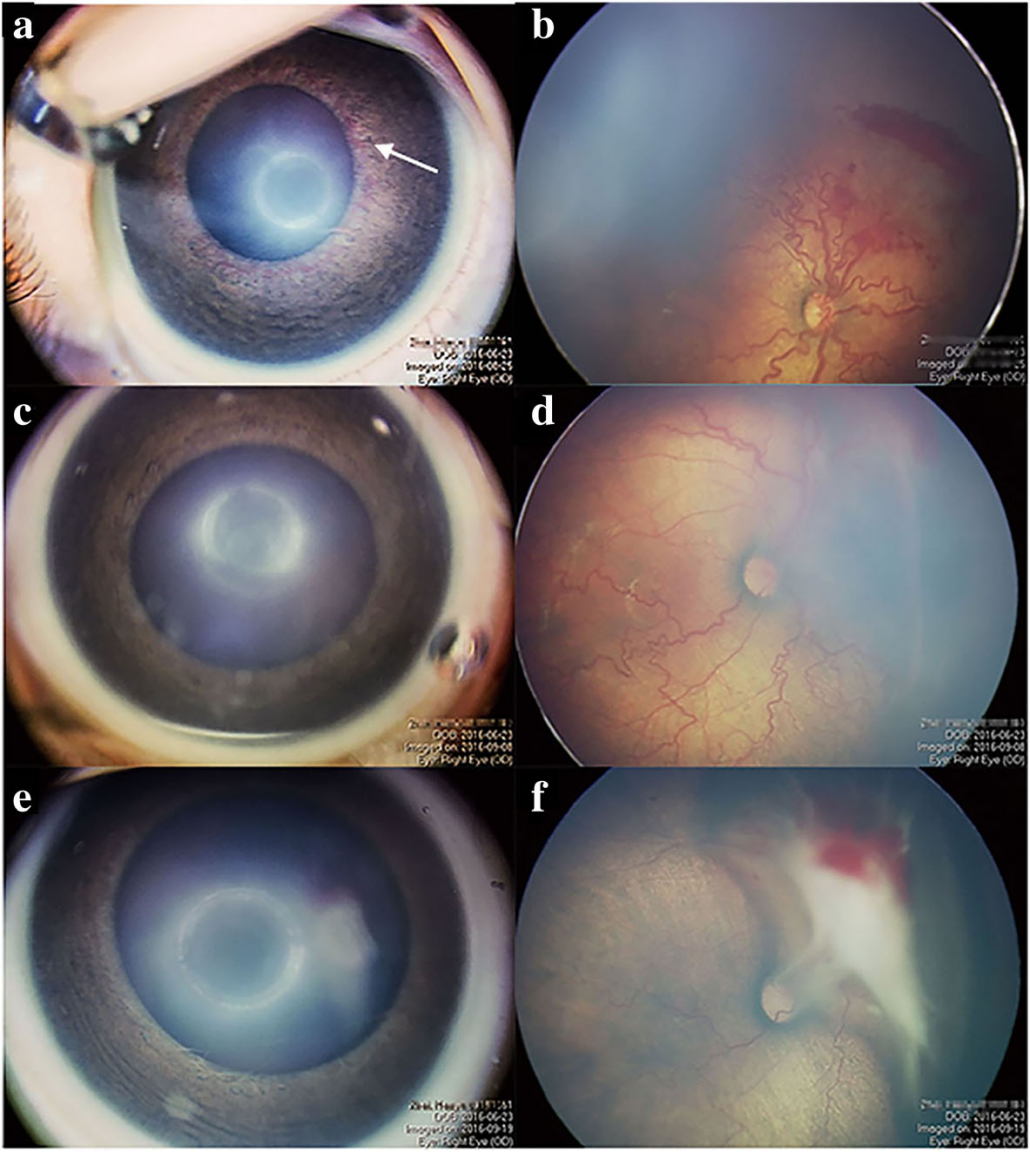
Fundus photographs of a patient in the retinal detachment group. **a** An eye of an infant with aggressive posterior retinopathy of prematurity (APROP) before intravitreal ranibizumab (IVR) treatment. The iris neovascularizations were observed clearly. **b** A fundus photograph of the same eye before IVR in which retinal hemorrhage and thick preretinal hemorrhage can be seen. **c** The iris neovascularizations regressed after two weeks of IVR. **d** Fibrosis is seen in the fundus after two weeks of IVR. **e** No iris neovascularizations were observed after 25 days of IVR. **f** Then, 25 days after the first IVR, tractional retinal detachment occurred

Table 2 shows the univariate logistic regression analysis of risk factors between the retinal detachment subgroup and no retinal detachment subgroup. Larger PMA was significantly more common in infants with retinal detachment. The other significant factors for retinal detachment included neutrophil count, GA and NRDS. Figure 1 shows an eye diagnosed with APROP treated with IVR that progressed to tractional retinal detachment. Retreatment before retinal detachment occurrence in the retinal detachment subgroup included IVR (12, 34.2%), laser (4, 11.4%), and IVR combined with laser (4, 11.4%). Retreatments in the non-retinal detachment subgroup included IVR (35, 28%), laser (29, 23.2%), and IVR combined with laser (18, 14.4%). Three dummy variables were created as dependent variables according to the method of retreatment.

**Table 2:** Univariate logistic regression analysis of retinal detachment and non-retinal detachment

Factors	Retinal detachment *N* = 35	Non-retinal detachment *N* = 125	OR	95% CI	*P* value
GA (weeks)	30.7 ± 2.0	29.8 ± 2.0	1.249	1.037,1.505	0.019
BW (g)	1376 ± 374	1330 ± 323	1.000	0.999,1.002	0.472
PMA (weeks)	38.3 ± 2.0	36.2 ± 1.8	1.905	1.456,2.491	< 0.01
Gender (male/female)	18/9	50/21	1.526	0.702,3.317	0.286
Neutrophil count (*10^9^/L)	1.95 ± 1.08	2.66 ± 1.60	0.68	0.494,0.938	0.019
Lymphocyte count (*10^9^/L)	4.91 ± 1.39	4.84 ± 1.53	1.034	0.806,1.326	0.794
NLR, median (QAR)	0.37 (0.19,0.60)	0.49 (0.33,0.75)	0.322	0.091,1.137	0.078
INV, n (%)	9 (25.7)	43 (34.4)	0.66	0.284,1.534	0.334
Retinal hemorrhage, n (%)	17 (48.6)	43 (34.4)	1.801	0.843,3.846	0.129
NRDS (treated with mechanical ventilation and surfactant) n (%)	2 (5.7)	11 (8.8)	1.173	0.945,1.456	0.149
Retreatment methods					
NO, n (%)	15 (42.9)	43 (34.4)			0.423
IVR, n (%)	12 (34.2)	35 (28.0)	1.570	0.458,5.384	0.473
Laser, n (%)	4 (11.4)	29 (23.2)	1.543	0.435,5.474	0.502

**Fig. 2:**
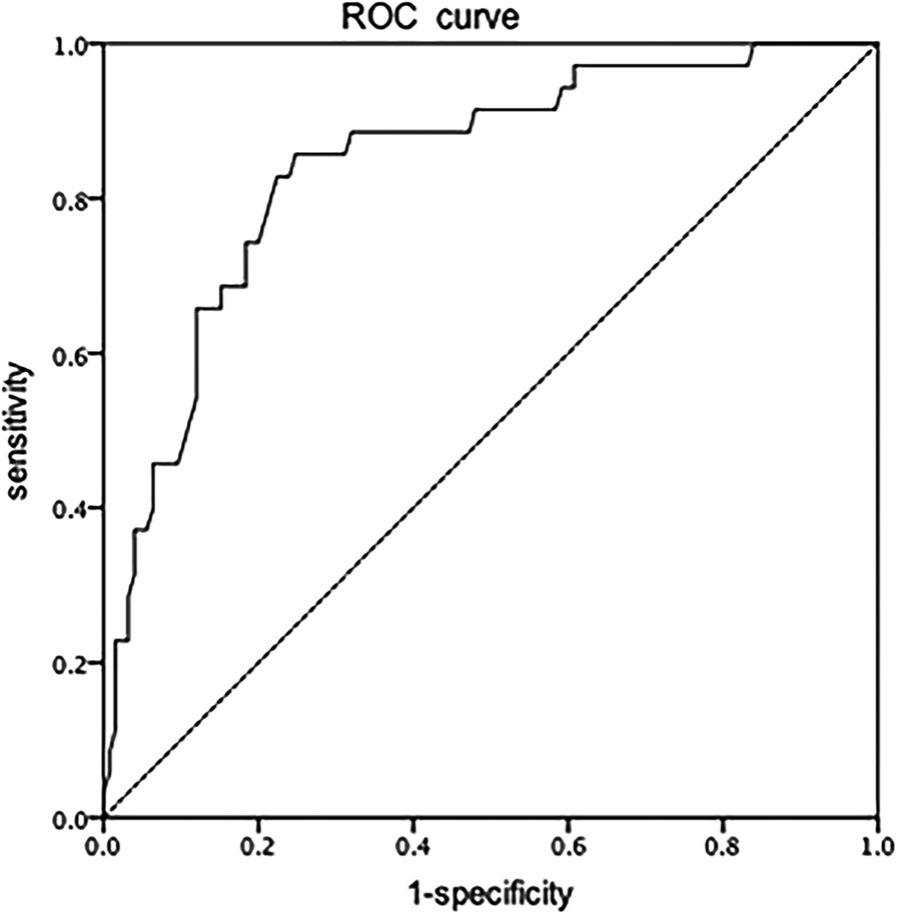
The receiver operating characteristic (ROC) curve for the risk factors postmenstrual age and neutrophil count at the first treatment. The area under the curve is 0.843 (95% CI: 0.770–0.916, *P* < 0.001)

Stepwise multiple logistic regression analysis was performed to identify risk factors that were significantly associated with retinal detachment in APROP. Variables that exhibited a significant correlation toward an association with retinal detachment in the univariate analysis (*P* < 0.2) included PMA, neutrophil count, GA and NRDS. These four variables were entered into a stepwise multiple logistic regression, which revealed that PMA at first treatment and neutrophil count were independent factors of retinal detachment in APROP treated with IVR (Table 3). A ROC curve was created for the stepwise logistic regression model, and the AUC was 0.843 (Fig. 2). The stepwise logistic regression model that included neutrophil count and PMA effectively predicted retinal detachment in APROP.

**Table 3:** Independent risk factors from the multivariate logistic regression analysis of the retinal detachment and non-retinal detachment subgroups

Variables in the multivariable model	Beta	Adjusted OR	95% CI	*P* value
PMA	0.719	2.052	1.524,2.761	< 0.001
Neutrophil count	−0.555	0.574	0.379,0.869	0.009

### Subgroup analysis: one injection and retreatment subgroups

**Fig. 3:**
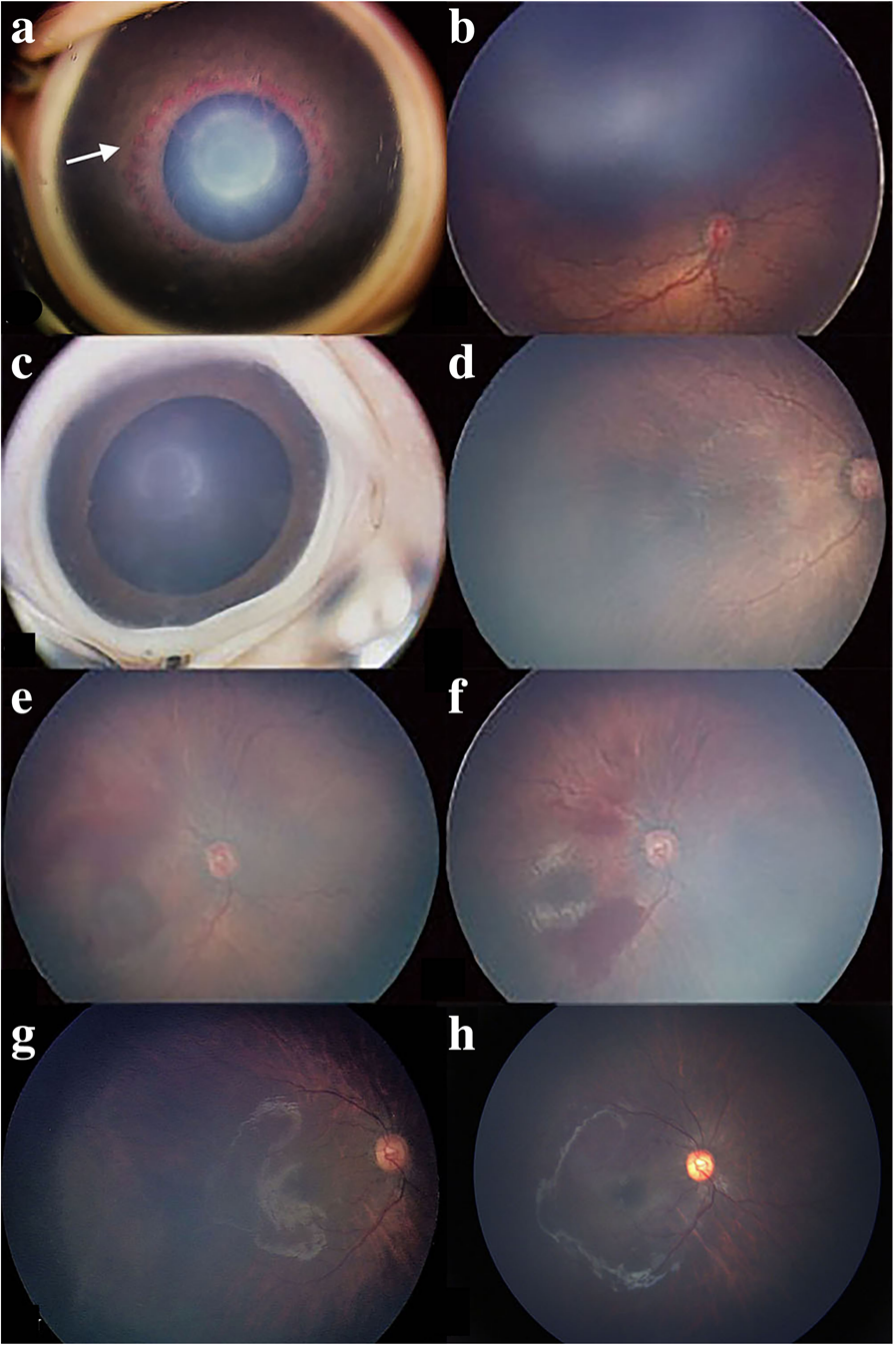
Photographs of a patient in the retreatment group before and after IVR treatment. **a** The anterior segment image of the right eye of an infant with aggressive posterior retinopathy of prematurity (APROP) before intravitreal ranibizumab (IVR) treatment showing iris neovascularizations around the pupil (white arrowhead). **b** A fundus photograph of the same eye before IVR. **c** Six weeks after the first IVR, the iris neovascularizations regressed completely. **d** Six weeks after the first IVR**,** the plus disease and ridge regressed. **e** The plus disease reoccurred, and vitreous retinal hemorrhage was observed after 12 weeks of the first IVR. The infant underwent a second IVR. **f** Four weeks after the second IVR, the plus disease regressed. **g** Thirteen months after the first IVR treatment. **h** Three years after the first IVR treatment

Forty-three (34.4%) of the 125 eyes without retinal detachment regressed after one injection, whereas 82 eyes (65.6%) required retreatment (Table 4). A total of 115 of the 125 eyes (92%) improved 1.5±1.2 weeks after the first IVR treatment. The mean recurrence period of APROP was approximately 7.5±6.9 weeks after the first IVR treatment. Univariate logistic regression analysis of the 82 eyes in the retreatment subgroup revealed that factors (*P* < 0.2 and 95% CI not including 1) associated with retreatment of APROP were BW (*P* = 0.053), GA (*P* = 0.048) and retinal hemorrhage (*P* = 0.009) (Table 5). These three factors were included in the stepwise multivariate regression analysis, and backward elimination identified retinal hemorrhage (*P* = 0.007) and BW (*P* = 0.04) as independent significant risk factors for the retreatment of APROP. Figure 3 shows an eye diagnosed with APROP that required retreatment after the first injection of ranibizumab.

**Table 4:** Univariate logistic regression analysis of the one injection and retreatment subgroups

Factors	One injection subgroup *N* = 43	Retreatment subgroup *N* = 82	OR	95% CI	*P* value
GA (weeks)	30.6 ± 2.3	29.5 ± 2.1	0.829	0.689,0.998	0.048
BW (g)	1457 ± 418	1289 ± 296	0.999	0.998,1.0	0.053
PMA (weeks)	36.6 ± 2.1	36.09 ± 1.78	0.919	0.745,1.134	0.433
Gender (male/female)	20/8	36/15	1.012	0.453,2.259	0.976
Neutrophil count (*10^9^/L)	2.77 ± 1.56	2.56 ± 1.63	0.894	0.712,1.122	0.333
Lymphocyte count (*10^9^/L)	4.59 ± 1.52	4.94 ± 1.53	1.139	0.885,1.466	0.31
NLR, median (QAR)	0.51 (0.36,0.80)	0.480 (0.272, 0.698)	0.536	0.204,1.41	0.206
INV, n (%)	13 (30.2)	30 (36.6)	1.331	0.604,2.936	0.478
Retinal hemorrhage, n (%)	8 (18.6)	35 (42.7)	3.258	1.346,7.886	0.009
NRDS (treated with mechanical ventilation and surfactant) n (%)	5 (11.6)	6 (7.3)	0.77	0.205,2.889	0.698

**Table 5:** Multivariate logistic regression analysis of the one injection and retreatment subgroups

Variables in the multivariable model	Beta	Adjusted odds ratio	95% CI	*P* value
Retinal hemorrhage	1.253	3.500	1.413,8.670	0.007
Birth weight	−0.001	0.999	0.997,1.000	0.04

## Discussion

This study demonstrated that 125 of 160 eyes (78.1%) exhibited good anatomic outcomes without tractional retinal detachment. Thirty-five eyes (21.9%) developed retinal detachment. Forty-three of the 160 eyes (26.9%) regressed after one injection, and 82 eyes (51.3%) required retreatment, with good anatomic outcomes. These results suggest that IVR is an effective treatment for APROP. Multivariate logistic analysis found that PMA and neutrophil count were independent factors for APROP development to tractional retinal detachment, and GA and retinal hemorrhage were independent factors for APROP recurrence that required retreatment.

Pradhan et al. [14] showed that retinal detachment occurred in 2 of 52 eyes (3.8%) with APROP after IVB monotherapy and that the mean PMA at IVB treatment was 35.11 weeks. Yetik et al. [15] conducted a study of 62 eyes from 31 infants with APROP treated by IVB and reported that the mean PMA at the first IVB treatment was 33.97 ± 1.47 (34.00) weeks and that no retinal detachment occurred. Similarly, in our study, the 39 eyes from the 21 patients whose PMA at the first IVR injection was ≤35.4 weeks also did not progress to retinal detachment. However, Zhao et al. [16] reported that retinal detachment occurred in 5 of the 49 eyes (10.2%) with APROP in patients given the first IVR injection at a mean PMA of 37.3 ± 5.2 weeks.

In our study, 35 of the 160 eyes (22%) progressed to retinal detachment. The differences between the previous reports and our study may be related to the late IVR treatment and high proportions of APROP with an older PMA. We found that the mean PMA at the first treatment was older in the retinal detachment subgroup (38.3 ± 2.0 weeks) than in the non-retinal detachment subgroup (36.2 ± 1.8 weeks). An older PMA indicated that the delayed IVR treatment was late. Yoshihiro et al. reported that infants who were injected at an older PMA progressed to tract retinal detachment more quickly [17]. A single burst of VEGF contributes to neovascularization during the ROP process [18], and late IVR treatment during the period when the VEGF levels are decreasing may induce fibrosis and contraction of flat neovascularization peripherally to cause tight detachment [16, 17]. Our study showed high proportions of APROP patients with older PMAs, because our hospital is a tertiary referral center in Beijing, and we receive patients with severe ROP from all parts of the country. In addition, because ranibizumab has a shorter half-life and lower molecular weight than bevacizumab [19, 20], the optimal effective dose of ranibizumab for APROP should be studied in the future.

Ahn et al. reported that compared with infants with non-APROP, infants with APROP exhibited significantly lower mean GA and weight at birth (*P* = 0.019, *P* < 0.001, respectively) and required more intense laser treatment with a higher retreatment rate [13]. Lower GA and BW were also risk factors for the development of APROP in our study. Infants with APROP exhibited extremely low BWs (1340 ± 334 g) and very early GAs (30.0 ± 2.0 weeks) in our study, which was consistent with the results of previous studies. However, we found that GA and BW were not associated with tractional retinal detachment in APROP.

The infants in this study who developed tractional retinal detachment exhibited an older PMA and lower neutrophil counts. Preterm infants with APROP exhibited a younger GA, and the older PMA suggested it was too late to recognize ROP. Indeed, this disease may progress in a relatively short time without early treatment. Thus, infants that miss the chance for early treatment may suffer tractional retinal detachment. As shown in the image of the patient’s eye in Fig. 1, retinal and preretinal hemorrhage were visible before treatment, which showed that the disease was severe. The baby was treated by IVR at a PMA of 38.6 weeks, which was too late. The vessel tortuosity did not obviously improve after two weeks of IVR treatment, and retinal detachment ultimately occurred.

This study is the first to report a lower neutrophil count as an independent risk factor for tractional retinal detachment in APROP. Fibrovascular membranes occur as epiretinal tissue during the retinal ischemic microangiopathy process, which may cause retinal detachment via traction mechanisms. Preretinal membranes in ROP are composed of collagen, myofibroblasts, fibroblasts, vascular endothelial cells, macrophages and other inflammatory cells [21]. Myofibroblasts in the preretinal membranes are contractile cells that exhibit features of fibroblasts and smooth muscle cells, which can lead to retinal traction in proliferative retinal diseases [22]. The presence of myofibroblasts in preretinal membranes and contraction are the pathogenic factors associated with tractional retinal detachment [23]. VEGF and transforming growth factor (TGF-beta) concentrations are significantly elevated in the vitreous of stage 4 ROP eyes, whereas the levels of other angiogenic factors are normal [24]. Anti-VEGF treatment successfully reduces pathological angiogenesis but does not decrease the proliferative changes associated with tractional retinal detachment [24]. TGF-beta 1 mediates cell contraction, which produces the tractional force that contributes to fibrotic diseases of the eye [25]. The TGF-beta inhibitors decorin and LY-364947 effectively reduced fibrosis and tractional retinal detachment in experimental rabbit models [26, 27]. Therefore, VEGF and TGF-beta may contribute to tractional retinal detachment.

Neutrophils exert an anti-angiogenic effect. Angiostatin K1–3 production by activated neutrophils inhibits basic fibroblast growth factor (FGF2) and VEGF in vitro and suppresses the angiogenesis induced by VEGF and FGF2 in vivo in a dose-dependent manner [28]. Neutrophil elastase (NE) is directly released from the granules of activated neutrophils, and this enzyme is responsible for the generation of angiostatin K1–3 [28]. NE degrades potent angiogenic factors, such as bFGF and VEGF, in a time- and concentration-dependent manner, which leads to loss of their angiogenic activity [29]. Therefore, a greater number of neutrophils may necessitate larger doses of angiostatin K1–3 and NE to produce stronger anti-VEGF effects.

Vascular endothelial growth factor receptor-1 (VEGFR-1) on neutrophils inhibits VEGF-mediated pathological angiogenesis. Sara et al. detected the VEGFR1 but not the VEGFR2 protein on circulating neutrophils from the blood of healthy volunteers using flow cytometry [30]. Luethy et al. reported significantly increased plasma sVEGFR1 concentrations in patients with various hematological and oncological malignancies after autologous stem cell transplantation and confirmed that neutrophils were the major source of VEGFR1 [31]. VEGF-A binding to VEGFR2 mediates pathological angiogenesis [32]. Moreover, VEGFR1 binds VEGFA with higher affinity than VEGFR2 [33], and VEGFR1 functions as a ‘trap’ for VEGFA by preventing VEGFA from binding to VEGFR2, which inhibits angiogenesis via the VEGFA/VEGFR2 signaling pathway. Therefore, VEGFR1 expressed by neutrophils is an endogenous anti-angiogenic factor.

However, Morgan et al. [34] reported that activated human neutrophils released VEGF, which played an important role in mediating the angiogenesis and new blood vessel formation processes in response to acute inflammatory reactions. Furthermore, neutrophil secretion of pro- or anti-angiogenic molecules may be related to environmental stimuli. Thus, neutrophils may represent an anti-angiogenic factor during APROP.

Previous studies have demonstrated that TGF-beta stimulates fibrosis and tractional retinal detachment in retinopathy. TGF-beta promotes neutrophil apoptosis and reduces the number of neutrophils via induction of IL-6 production from mast cells [35]. Thus, the lower number of neutrophils in the tractional retinal group may have resulted in apoptosis promotion due to the high concentration of TGF-beta. Previous studies have also supported anti-TGF-beta as an effective therapeutic method to reduce tractional retinal detachment. However, serum TGF-beta concentrations were not obtained from the patients. This study was the first report to identify the number of neutrophils as a predictive factor for retinal detachment in ROP.

A younger GA was not related to APROP recurrence or retreatment. A younger GA indicates a longer duration of exposure to adverse postnatal insults, and the maturation of retinal vasculature in infants requires more time in the absence of the factors that normally occur in the intrauterine environment. This environment may increase the probability of the appearance of ROP. Gaurav et al. reported that 4 infants (9.1%) were born after 32 weeks of gestation in a retrospective review of 81 eyes from 44 consecutive Indian preterm infants with APROP [36]. Therefore, APROP occurred in more mature babies. A low BW was a risk factor for APROP recurrence and retreatment in our study, which was consistent with a previous study performed in the US [37].

Retinal hemorrhage, which is correlated with the ROP severity, is significantly related to APROP recurrence and retreatment. The immature retinal vasculature, which lacks structural support from smooth muscles, collagen, pericytes and elastin, is more fragile than mature vessels and may be susceptible to rupture. Kim et al. [38] reported that retinal hemorrhage of ROP was a risk factor for worsening of ROP. Another previous study reported that the presence of retinal hemorrhage was significantly associated (*P* < 0.0001) with the presence and severity of ROP [39], which was consistent with our study results.

### Study limitations

The retrospective nature of this study and the lack of a control group warrant further research to reduce tractional retinal detachment and recurrence in APROP. Nevertheless, our study identifies independent factors associated with retinal detachment in APROP and independent factors related to APROP recurrence and retreatment following initial IVR treatment. This study is also the first to show that a low neutrophil number is an independent predicting factor for retinal detachment in APROP. More robust data and randomized trials are needed to evaluate the benefits and clinical use of the neutrophil count as a prognostic factor for APROP. Additionally, optimal cut-off levels need to be established for neutrophil counts. Other remaining problems for the treatment of APROP, such as the visual and refractive outcomes and the optimal injection dose, are high priorities for future research.

## Conclusion

In summary, IVR is an effective treatment for APROP. The factors associated with retinal detachment and recurrence should be noted, and vigilant follow-up is needed to ensure timely retreatment.

## Abbreviations

APROP: Aggressive posterior retinopathy of prematurity
BW: Birth weight
GA: Gestational age
IVR: Intravitreal ranibizumab
NLR: Neutrophil-to-lymphocyte ratio
PMA: Postmenstrual age
ROP: Retinopathy of prematurity
VEGF: Vascular endothelial growth factor
